# Treating Fasciotomy Wounds with Negative Pressure Wound Therapy with Instillation and Dwell Time (NPWTi-d)

**DOI:** 10.7759/cureus.852

**Published:** 2016-10-28

**Authors:** Priscilla Lee

**Affiliations:** 1 Vascular Surgery, UCLA Medical Center

**Keywords:** fasciotomy, wounds, negative pressure wound therapy

## Abstract

Acute compartment syndrome (ACS) is a serious complication of lower-extremity trauma caused by accidents or post-procedure complications. ACS is characterized by increased pressure within the compartment, resulting in reduced blood flow, tissue hypoxia, and tissue necrosis. Fasciotomies to relieve pressure and debridement of necrotic tissue comprise primary treatment. My purpose is to present initial experience using negative pressure wound therapy with instillation and dwell time (NPWTi-d)* to treat fasciotomy wounds in two patients. NPWTi-d provides automated, volumetric control of instilled topical wound solutions with a dwell time in combination with negative pressure wound therapy (NPWT).

Patient 1, a 33-year-old male injured in a motorcycle accident, developed ACS within 24 hours of hospitalization. Prior treatments included wet-to-dry dressings and NPWT^†^. In the latter course of treatment, NPWTi-d was applied; 40 ml of normal saline (NS) were instilled with a ten-minute dwell time, followed by four hours of NPWT at ‑125 mmHg. After five days of NPWTi‑d, granulation tissue covered the bone. Four days later, the patient was discharged home. The wound continued to improve and, at the last recorded visit, was completely closed.

Patient 2, a 44-year-old male, developed right lower extremity ACS due to complications post cardiac surgery. NPWT was initiated in the hospital and continued post-discharge to a nursing home. The patient was readmitted to the hospital with a right leg wound infection that was surgically debrided. NPWTi-d was then applied; 60 ml of NS were instilled with a ten-minute dwell time, followed by 3.5 hours of NPWT at -125 mmHg. After ten days of NPWTi-d, granulation tissue covered the bone. In Patient 2, NPWTi-d improved the likelihood of healing in a malnourished patient who had been critically ill by promoting granulation tissue over exposed bone. The use of NPWTi-d with NS contributed to positive outcomes for both patients.

*V.A.C. VeraFlo™ Therapy, ^†^V.A.C.® Therapy (KCI, an Acelity company, San Antonio, TX)

## Introduction

Acute compartment syndrome (ACS) is a serious complication of the lower extremity caused by trauma or surgical complications. ACS is characterized by increased pressure within the compartment, resulting in reduced blood flow, tissue hypoxia, and tissue necrosis [1]. A fasciotomy to relieve pressure and debridement of necrotic tissue comprise the primary treatment. The resulting wound is at risk of developing an infection or other complications. Negative pressure wound therapy with instillation and dwell time (NPWTi-d) is a treatment for selected complex wounds. NPWTi-d was found to be an effective treatment modality for specific patients [2].

The purpose of these case studies is to present initial experience using NPWTi-d to treat fasciotomy wounds in two patients. Both patients met the panel recommendations for appropriate use of NPWTi-d of wound complexity and appropriate patient. Prior to the catastrophic events that necessitated hospital admission and prolonged ICU stays, both patients were healthy males with active lifestyles and no significant comorbidities. Both sustained complex wounds as defined by Kim et al [3]. NPWTi-d provides intermittent delivery of a timed, predetermined volume of topical wound solution, which is allowed to dwell in the wound bed for a user-selected time period prior to resuming NPWT [4]. Negative pressure wound therapy with instillation has been used effectively to assist in granulation in acute, subacute, and chronic wounds [5-8].

NPWTi-d has been shown to be an effective treatment modality in treating patients with post-traumatic osteomyelitis of the pelvis/leg. In each of these case studies, normal saline was instilled into the fasciotomy wounds of both patients, who also received antibiotic treatment for osteomyelitis [9].

**Figure 1:**
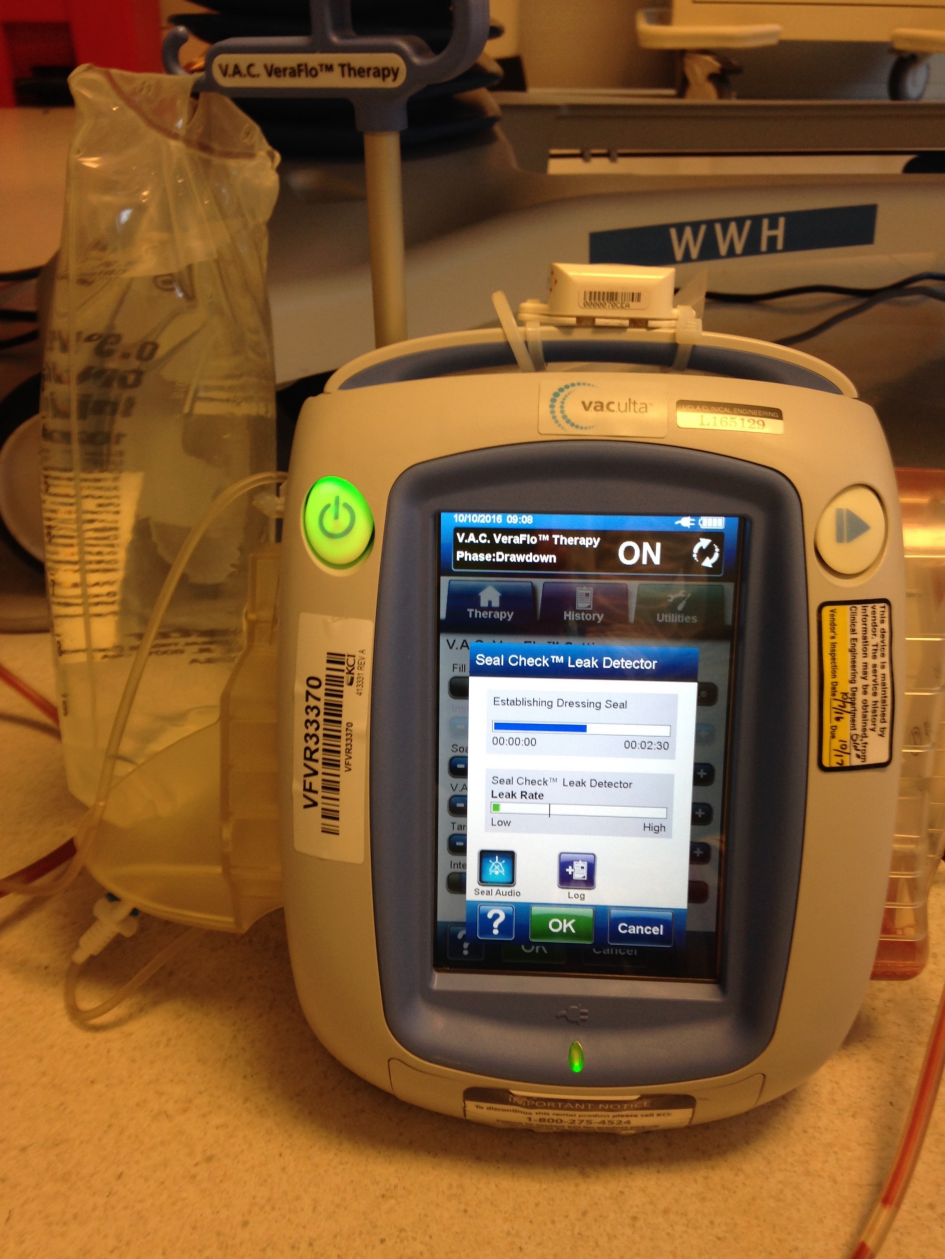
VAC VeraFlo

Figure 1 shows the VAC VeraFlo.

## Case presentation

### Case study 1

**Figure 2:**
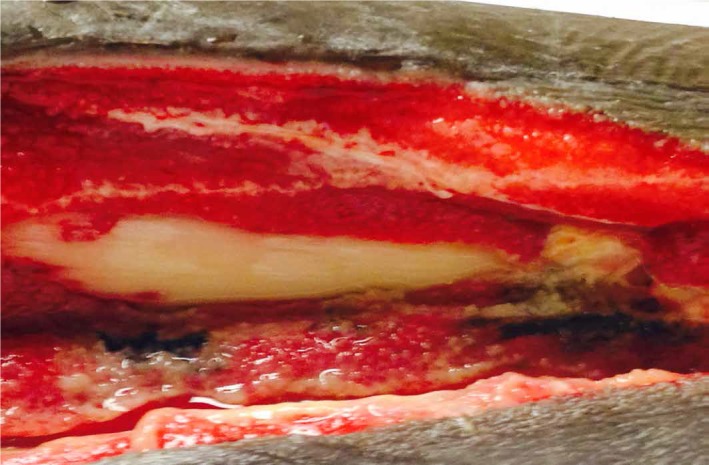
Exposed Bone Prior to NPWTi-d with Normal Saline

A 33-year-old male injured in a motorcycle accident developed lower-extremity ACS within 24 hours of hospitalization. Wet-to-dry dressings were used over the fasciotomy wounds initially, followed by NPWT†. NPWTi-d was applied to the right lower extremity which had exposed bone (Figure 2).

†V.A.C.® Therapy (KCI, an Acelity company, San Antonio, TX)

**Figure 3:**
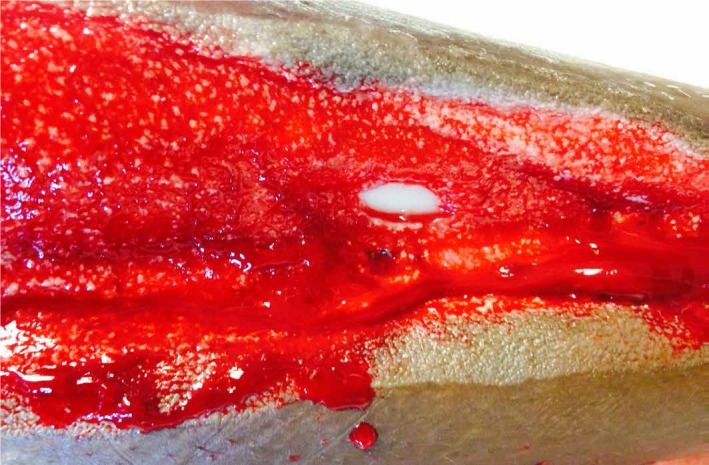
Bone Coverage after Three Days of NPWTi-d Instillation of 40 ml of NS with a ten-minute dwell time, followed by four hours of NPWT at -125 mmHG.

After 40 ml of normal saline (NS) were instilled with a ten-minute dwell time, followed by four hours of NPWT at -125 mmHg for a period of three days, the amount of exposed bone was noticeably decreased (Figure 3).

**Figure 4:**
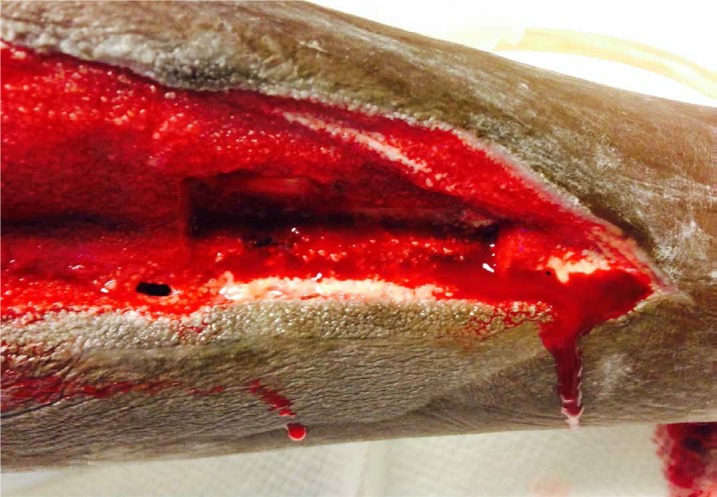
Complete Bone Coverage after Five Days of NPWTi-d

After five days of NPWTi-d, granulation tissue covered the bone (Figure 4).

**Figure 5:**
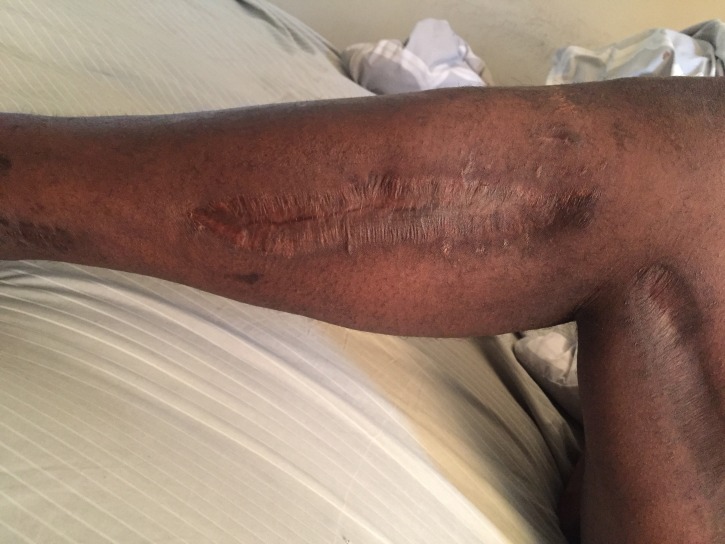
Wound Appearance One Year Later Case 1 The wound is completely healed with VAC VeraFlo alone and no further surgical intervention. The patient has returned to his job as a personal trainer.

Four days later, the patient was discharged home. Figure 5 shows the healing one year later.

### Case study 2

**Figure 6:**
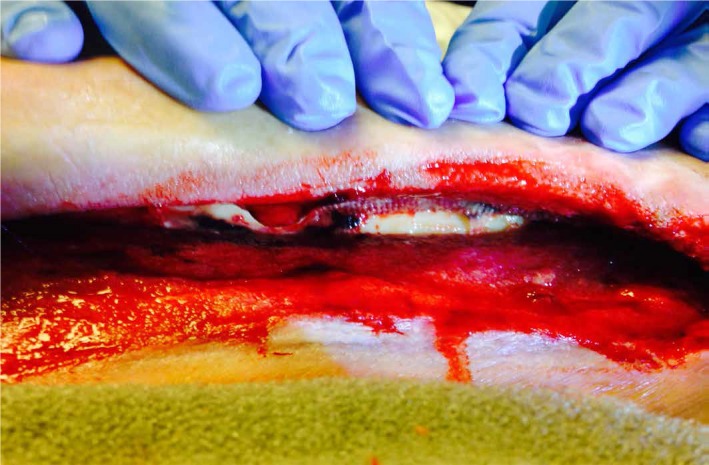
Wound with Exposed Bone Post-Debridement

A 44-year-old male developed right lower extremity ACS due to multiple complications post-cardiac surgery. NPWT was initiated on the fasciotomy wound in the hospital and continued for three days post-discharge to a nursing home. A month later, the patient was readmitted to the hospital with an infection in the right leg wound which was then surgically debrided (Figure 6).

**Figure 7:**
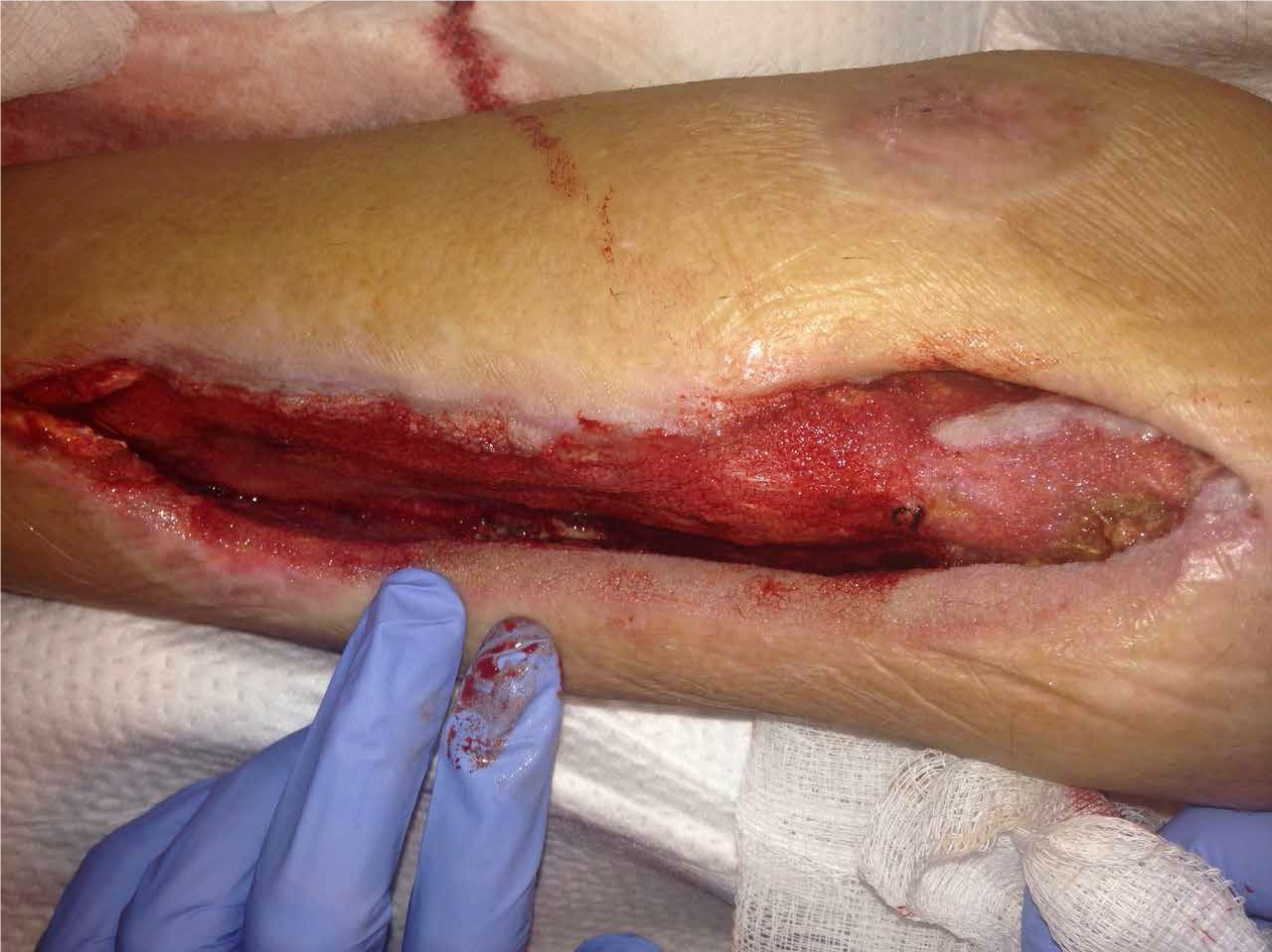
Bone Coverage after Five Days of NPWTi-d Instillation of 60 ml of NS with a ten-minute dwell time, followed by 3.5 hours of NPWT at -125 mmHg.

Oral antibiotics were prescribed for osteomyelitis. NPWTi-d was applied; 60 ml NS was instilled with a ten-minute dwell time, followed by 3.5 hours of NPWT at -125 mmHg (Figure 7).

**Figure 8:**
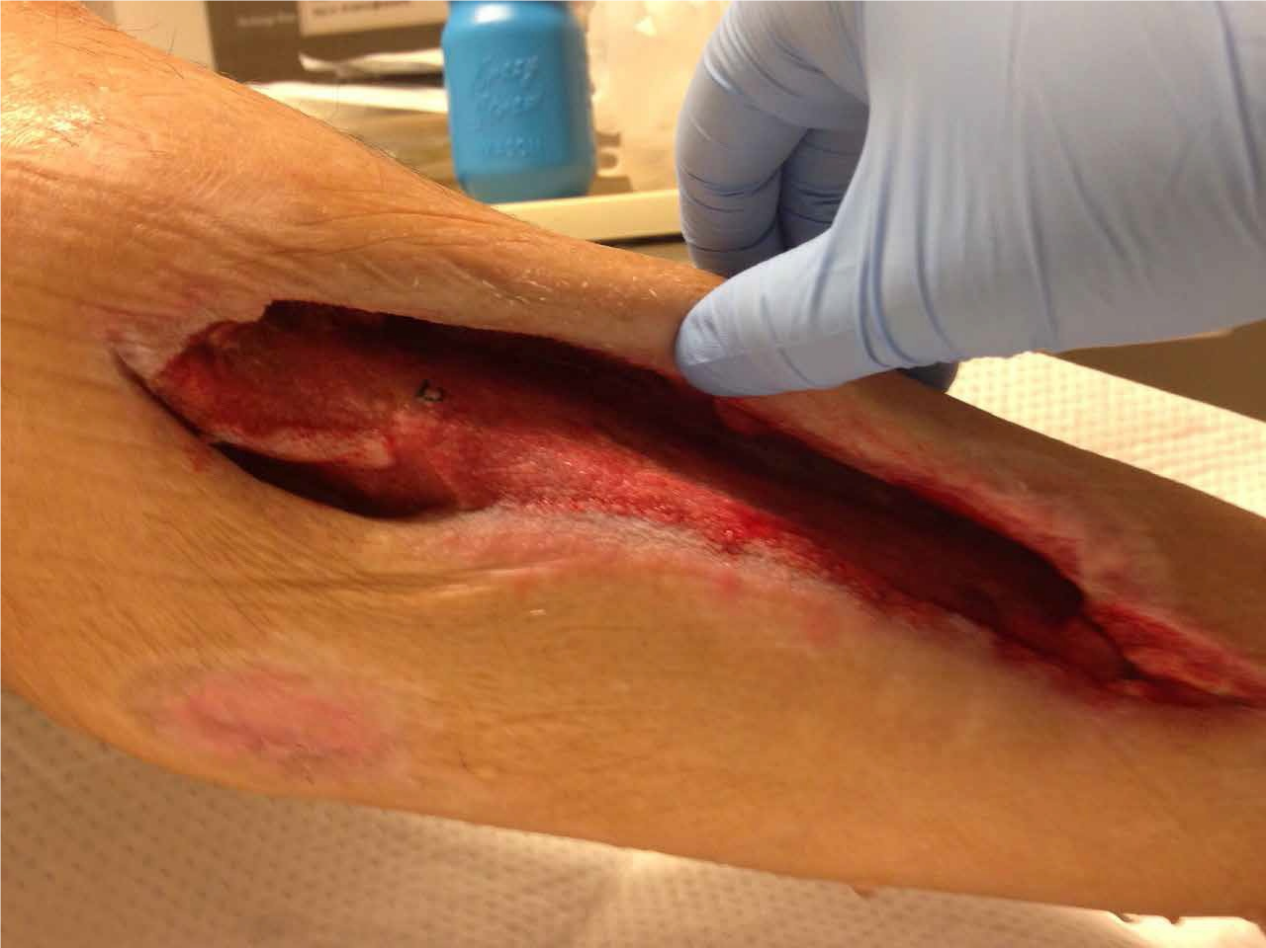
Complete Bone Coverage after Ten Days of NPWTi-d

After ten days of NPWTi-d, granulation tissue completely covered the bone (Figure 8).

**Figure 9:**
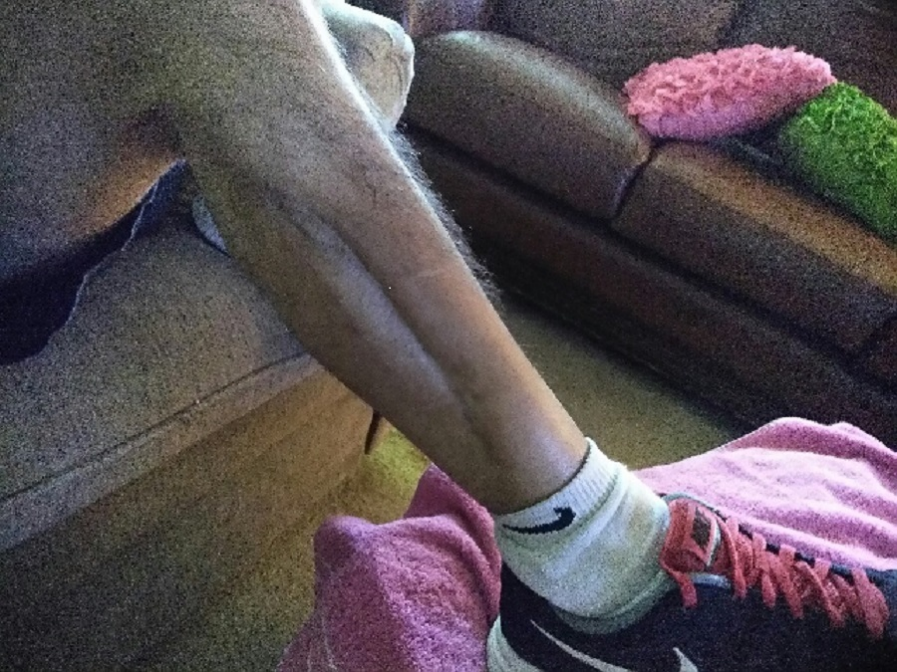
Wound Appearance One Year Later Case 2 Wound completely healed one year later with VAC VeraFlo alone and no further surgical interventions. The patient is wearing running shoes because he is about to compete in a local 5K.

The patient was ultimately discharged home on oral antibiotics to complete the course of treatment for osteomyelitis. Figure 9 shows the healing one year later.

The patients agreed to participate and were explained the nature and objectives of this study, and informed consents were formally obtained. No references to the patients' identities were made at any stage during data analysis or in the report.

## Discussion

Unless diagnosed quickly, ACS can result in tissue necrosis as a result of intracompartmental ischemia and hypoxia. Fasciotomies and debridement can relieve the pressure. However, in compromised patients, these wounds can develop an infection (especially osteomyelitis). Depending on therapy goals, NPWTi-d can be used to cleanse the wound, promote granulation tissue formation, or a combination of both [10].^ ^

In these two patients, NPWTi-d with NS was used for both cleansing and granulation tissue formation, and was effective in producing bone coverage in a short time (five days for Patient (Case) 1 and ten days for Patient (Case) 2). 

### Case study 1

The first patient is a 33-year-old male who was in a motorcycle accident September 2014. He sustained an open right tibia-fibula fracture and then underwent right, above-knee, popliteal-to-posterior-tibial-artery bypass with reversed left great saphenous vein graft, right lower extremity four quadrant fasciotomies, and reduction/nailing of the tibia and femoral shaft.  He developed a wound infection, and wound vacs were placed on the medial and lateral fasciotomy sites. He was sent home after a 45-day hospital stay. He was discharged home with a wound vac. When he followed up one week after discharge in the Hyperbaric Medicine Clinic, it was noted that he had necrotic tissue in the lateral fasciotomy site. He was taken to the operating room twice on October 16 and October 27 to debride the necrotic tissue and further extend the fasciotomy incision. At the surgery on October 27, the fasciotomy was extended, and debridement was to the level of the bone. There were 4 cms of exposed bone in the wound. The lateral compartment at that time measured 23 cm x 6 cm x 3 cm. The patient expressed considerable depression and frustration at another prolonged hospital stay; he was anxious to return home. In an effort to accelerate the granulation tissue and return the patient home as soon as possible, a VAC VeraFlo was applied on October 31. Forty milliliters of normal saline were used to irrigate the wound for ten minutes every four hours. This was the first time the VAC VeraFlo had ever been used in our hospital, and there were no established guidelines yet for instillation and dwell time. On November 5, 2014, there was 1 cm x 1.5 cms of exposed bone in the wound. On November 7, 2014, there was granulation tissue over bone. The patient was discharged home on November 8, 2014, on oral antibiotics. Home Health was arranged to apply wound vacs in the home, and this was continued until the wound healed approximately six weeks later.

One year post-treatment, the patient is planning his wedding and has resumed his job as a personal trainer.

### Case study 2

The second patient is a 44-year-old male who presented to the emergency room in January 2015 with an aortic dissection from the sternal notch to the bifurcation of the aorta. Forty-eight hours later, he developed bilateral lower extremity compartment syndrome requiring decompressive, four-compartment fasciotomies of the right and lower extremities. A wound vac was placed in the operating room. Amputation due to poor motor control was discussed with the patient and his mother. They requested that we “do everything” to save his legs. He had a prolonged and very complicated hospitalization with resulting end-stage-renal disease on hemodialysis at discharge, blindness due to posterior ischemic optic neuropathy or central retinal artery occlusion. He required hand grafts for upper extremity fasciotomies. He developed a pseudomonas infection of his G-tube site. He developed a Stage 2 pressure ulcer on his occiput. He developed ischemic bowel s/p colectomy/partial small bowel resection/ileostomy as well as a circulatory arrest. He had postoperative respiratory failure requiring a tracheostomy. A right and left pneumothorax required chest tubes. He was discharged to a skilled nursing facility in May 2015 with wound vacs to bilateral lower fasciotomy sites. He was admitted to a shared room with a patient on contact precautions. He had been walking with a front wheeled walker but had to stop due to leg pain. The wound vac was removed and wet-to-dry dressings applied. Three weeks later, he was admitted to our hospital with sepsis due to wound infections. Wound cultures grew klebsiella pneumonia and enterococcus species. Further debridement was required. The patient was eager to resume his rehabilitation and return home given his previous prolonged hospital stay. The VAC VeraFlo was applied in an effort to hasten granulation tissue and his return home. The patient was discharged three weeks later to his home, with granulation tissue over bone, on oral antibiotics, and with a wound vac, and under the care of his mother and sister.

One year later, the patient is off dialysis and has had his colostomy reversed. Although he is blind, he has competed in several local 5K running races.

## Conclusions

The use of NPWTi-d with NS contributed to positive outcomes for both patients. These included reduction in the length of stay, successful wound healing, and returning to the previous level of functioning. Despite consideration of amputation of the limbs of both of these patients, both have returned to active lifestyles which include personal training and competing in running events.
